# Copy Number Variation of Satellite III (1q12) in Patients With Schizophrenia

**DOI:** 10.3389/fgene.2019.01132

**Published:** 2019-11-22

**Authors:** Elizaveta S. Ershova, Oksana N. Agafonova, Natalia V. Zakharova, Lidia V. Bravve, Elizaveta M. Jestkova, Vera E. Golimbet, Tatiana V. Lezheiko, Anna Y. Morozova, Andrey V. Martynov, Roman V. Veiko, Pavel E. Umriukhin, Georgiy P. Kostyuk, Sergey I. Kutsev, Natalia N. Veiko, Svetlana V. Kostyuk

**Affiliations:** ^1^Department of Molecular Biology, Research Centre for Medical Genetics, Moscow, Russia; ^2^I.M. Sechenov First Moscow State Medical University, Moscow, Russia; ^3^Moscow Healthcare Department, N. A. Alexeev Clinical Psychiatric Hospital №1, Moscow, Russia; ^4^Moscow Healthcare Department, P.B. Ganushkin Clinical Psychiatric Hospital №4, Moscow, Russia; ^5^Department of Clinical Genetics, Mental Health Research Center, Moscow, Russia; ^6^Department of Basic and Applied Neurobiology, V. Serbsky National Medical Research Center for Psychiatry and Narcology, Moscow, Russia; ^7^P.K. Anokhin Institute of Normal Physiology, Moscow, Russia

**Keywords:** CNV, satellite III, 1q12, schizophrenia, hypoxia, ROS

## Abstract

**Introduction:** It was shown that copy number variations (CNVs) of human satellite III (1q12) fragment (f-SatIII) reflects the human cells response to stress of different nature and intensity. Patients with schizophrenia (SZ) experience chronic stress. The major research question: What is the f-SatIII CNVs in human leukocyte as a function of SZ?

**Materials and Methods:** Biotinylated pUC1.77 probe was used for f-SatIII quantitation in leukocyte DNA by the non-radioactive quantitative hybridization for SZ patients (N = 840) and healthy control (HC, N = 401). SZ-sample included four groups. Two groups: first-episode drug-naïve patients [SZ (M-)] and medicated patients [SZ (M+)]. The medical history of these patients did not contain reliable confirmed information about fetal hypoxia and obstetric complications (H/OCs). Two other groups: medicated patients with documented H/OCs [hypoxia group (H-SZ (M+)] and medicated patients with documented absence of H/OCs [non-hypoxia group (NH-SZ (M+)]. The content of f-SatIII was also determined in eight post-mortem brain tissues of one SZ patient.

**Results:** f-SatIII in human leukocyte varies between 5.7 to 44 pg/ng DNA. f-SatIII CNVs in SZ patients depends on the patient’s history of H/OCs. f-SatIII CN in NH-SZ (M+)-group was significantly reduced compared to H-SZ (M+)-group and HC-group (p < 10^-30^). f-SatIII CN in SZ patients negatively correlated with the index reflecting the seriousness of the disease (Positive and Negative Syndrome Scale). Antipsychotic therapy increases f-SatIII CN in the untreated SZ patients with a low content of the repeat and reduces the f-SatIII CN in SZ patients with high content of the repeat. In general, the SZ (M+) and SZ (M-) groups do not differ in the content of f-SatIII, but significantly differ from the HC-group by lower values of the repeat content. f-SatIII CN in the eight regions of the brain of the SZ patient varies significantly.

**Conclusion:** The content of f-SatIII repeat in leukocytes of the most patients with SZ is significantly reduced compared to the HC. Two hypotheses were put forward: (1) the low content of the repeat is a genetic feature of SZ; and/or (2) the genomes of the SZ patients respond to chronic oxidative stress reducing the repeats copies number.

## Introduction

Recently, we found a significant variability of the fragment of the human satellite III (1q12) (f-SatIII) content in the blood leukocytes and cultured human fibroblasts (Ershova et al., 2019b). Human aging, genotoxic and social stresses significantly affect the variability of repeat content in the people. In response to stress, the f-SatIII content in the human genome may either increase or decrease. The response depends on the stress intensity and the genetic cells characteristics. We also hypothesized that the f-SatIII content in the human genome changes not only with aging or under environmental factors but also in diseases accompanied by oxidative stress.

The main goal of the new study was to test this hypothesis with an example of the chronic disease that is associated with oxidative stress. As the object of the study, we chose a chronic disease of schizophrenia (SZ). SZ is a mental disease found in about 1% of the population with 70–80% heritability (Cardno et al., 1999). Multiple genetic and environmental factors contribute to the etiology and pathogenesis of SZ.

Oxidative stress has been proposed as one of the possible reasons of SZ. Elevated reactive oxygen species levels and declined antioxidant statuses in the brain and peripheral tissues of the patients with SZ have been reported (Emiliani et al., 2014; Sertan Copoglu et al., 2015; Smaga et al., 2015; Hardingham and Do, 2016; Koga et al., 2016; Barron et al., 2017; Maas et al., 2017; Patel et al., 2017). In addition, patients with SZ experience severe social and emotional stress (Howes and Murray, 2014).

SZ is considered as a systemic disorder, which is associated with biochemical disturbances not only in the brain cells (Kirkpatrick, 2009). In recent years, there has been an intensive search for blood-based markers for SZ (Chan et al., 2011; Lai et al., 2016; Sabherwal et al., 2016; Perkovic et al., 2017).

We suggested that the sample of SZ patients, compared with healthy control, should be characterized by significant disproportion of f-SatIII content in the blood leukocytes. We observed such disproportion for mentally healthy individuals who experienced chronic oxidative stress due to long-term work with sources of low doses of IR (Korzeneva et al., 2016; Ershova et al., 2019b). The literature data also support this assumption. A number of authors pointed out that pathways controlling genome stability during brain development in the ontogeny are functionally variable and produce somatic genome variations unequally distributed across different tissues (Yurov et al., 2007; Piotrowski et al., 2008; Yurov et al., 2018). Genomic rearrangements and gene copy number alterations often are considered as the reasons for nervous system disorders (Lee and Lupski, 2006; Zhuo et al., 2017). Associations of the copy number variations (CNVs) in different human chromosomes with SZ were found (Bassett et al., 2010; Vassos et al., 2010; Kirov et al., 2014; Marshall et al., 2017; Zhuo et al., 2017; Kushima et al., 2018; Kendall et al., 2019; Warland et al., 2019). Increased level of clonal chromosome-specific mosaic aneuploidy (up to 4%) involving chromosomes 1 in SZ brain was uncovered (Yurov et al., 2001; Yurov et al., 2008).

To test the assumption that f-SatIII variability is increased in the SZ patients compared with healthy controls (HCs), we determined the f-SatIII content in leukocytes of 401 individuals of HC-group and 840 individuals with SZ. Contrary to our expectations, we found an unexpected effect—a decrease in the content of f-SatIII in SZ patients without prenatal hypoxia and obstetric complications (H/OCs) in anamnesis. The low f-SatIII content in the SZ patients DNA may be a genetic feature of that disease.

## Materials and Methods

### Population Samples

The dataset included 1,241 individuals (401 HC-group and 840 SZ-group) inhabiting Moscow and the Moscow region. Clinical and demographic characteristics of the groups are shown in **Table 1**.

**Table 1:** Demographic and clinical measures in the SZ patients and HC group.

Index	HC Year of the birth: 1935–2001 N = 401	SZ Year of the birth: 1935–2001 N = 840			
		N. A. Alexeev Clinical Psychiatric Hospital №1 of Moscow Healthcare Dep. N = 554		Mental Health Research Center N = 286	
		1	2	3	4
		SZ (M-) N = 283	SZ (M+) N = 271	H-SZ (M+) N = 143	NH-SZ(M+) N = 143
Age	38.1 ± 13.4	31.5 ± 11.2	37.1 ± 19.7	27.3 ± 8.1	29.2 ± 8.4
Age of SZ onset		20.6 ± 7.4	19.4 ± 8.7	17.2 ± 6.8	17.6 ± 3.9
Age of SZ manifestation		25.0 ± 7.0	22.8 ± 8.2	21.0 ± 6.7	20.9 ± 5.4
PANSS		100.3 ± 25.7	97.3 ± 32.7	82.9 ± 22.4	79.1 ± 22.9
Gender (men/women)	250/151	457/383			
Weight (kg)	73.1 ± 16.2	70.6 ± 14.1			
Height (m)	1.75 ± 0.11	1.72 ± 0.08			
BMI (kg/m2)	23.6 ± 3.3	23.9 ± 4.3			
Never smoked (%)	63	51			
More than 20 cigarettes a day (%)	12	19			

Five hundred fifty-four blood samples were obtained from N. A. Alexeev Clinical Psychiatric Hospital №1 (CPH1) patients and 286 blood samples were obtained from the Mental Health Research Center (MHRC) patients. These two samples have different reliability of the fetal H/OCs.

One hundred forty-three SZ patients from MHRC have had documented H/OCs and 143 SZ patients from MHRC have had documented normal childbirth without any H/OCs. All the SZ patients from MHRC were treated with antipsychotics.

Although some patients from CPH1 or their relatives also reported possible H/OCs during childbirth, this fact was not documented and therefore was neglected in the analysis. The SZ patients from CPH1 were divided into two groups: 283 first-episode drug-naïve patients and 271 SZ patients with a long time antipsychotics therapy. In 93 first-episode drug-naïve patients blood sampling was performed twice—before the treatment and a month later after the standard course of antipsychotic therapy started. For the treatment of the acute disorders standard antipsychotics were used: haloperidol, ziprasidone, paliperidone, chlorpromazine, clozapine, risperidone, quetiapine, and olanzapine.

Psychopathology and functionality of patients were measured according to the Positive and Negative Syndrome Scale (PANSS) (Kay et al., 1987). Patients were diagnosed with paranoid SZ (F20.00 or F20.01) according to International Classification of Diseases 10 criteria using structured interviews (Mini-International Neuropsychiatric Interview). Diagnoses were also confirmed pursuant to Diagnostic and Statistical Manual of Mental Disorders, 4th Edition criteria.

The control group consisted of 401 subjects born in 1935 – 2001. These individuals were part of the general sample of mentally healthy individuals, which was described in detail in our previous study (Ershova et al., 2019b).

Brain tissue samples of the paranoid SZ patient (34-year-old male, cause of death—pulmonary embolism) were obtained in the V. Serbsky National Medical Research Center for Psychiatry and Narcology, Moscow.

***The patient consent to the various analyses performed.*** The investigation was carried out in accordance with the latest version of the Declaration of Helsinki and was approved by the Regional Ethics Committees of Research Centre for Medical Genetics, Hospital №1 of Moscow Healthcare Department (CPH1), and MHRC. All participants signed an informed written consent to participate after the procedures had been completely explained. The relatives of the deceased patient gave written permission to use his biological material for scientific purposes.

### Isolating of DNA From the Leukocytes

The leukocytes were isolated from 5 ml of blood by the method of Boyum (Boyum, 1968). Five milliliters of the solution containing 2% sodium lauryl sarcosylate, 0.04 M Ethylenediaminetetraacetic acid, and 150 µg/ml RNAse A (Sigma, USA) was added to the fresh leucocytes for 45 min (37°C) and then was treated with proteinase K (200 µg/ml, Promega, USA) for 24 h at 37°C. The lysate samples were extracted with an equal volume of phenol, phenol/chloroform/isoamyl alcohol (25:24:1), and chloroform/isoamyl alcohol (24:1), respectively. DNA was precipitated by adding 1/10 volume of 3 M sodium acetate (pH 5.2) and 2.5 volume of ice-cold ethanol. For the extraction procedure, only freshly distilled solvents were used. Phenol was stabilized with 8-hydroxyquinoline. Finally, the DNA was collected by centrifugation at 10,000×*g* for 15 min at 4°C, washed with 70% ethanol (v/v), dried, and dissolved in water.

### The Quantitation of the Purified Genomic DNA

The success of NQH depends on the accurate quantification of the DNA present in the samples. We perform quantification in a minimum of two different steps, called pre- and final quantification. The first one gives a rough estimate of the initial amount of DNA in each sample by the method of ultraviolet spectroscopy. At the end of the first step, the amount of DNA needed to make a 60 ng/ml solution of DNA is calculated. The final quantification uses this solution to calculate the exact amount of DNA needed to dilute samples to 50 ng/ml. The final DNA quantification is performed fluorimetrically using the PicoGreen dsDNA quantification reagent by Molecular Probes (Invitrogen, CA, USA). The assay displays a linear correlation between dsDNA quantity and fluorescence within a wide range of concentrations. The DNA concentration in the sample is calculated according to a DNA standard curve. We use EnSpire equipment (Finland) at excitation and emission wavelengths of 488 and 528 nm, respectively.

### Nonradioactive Quantitative Hybridization

**Figure 1:**
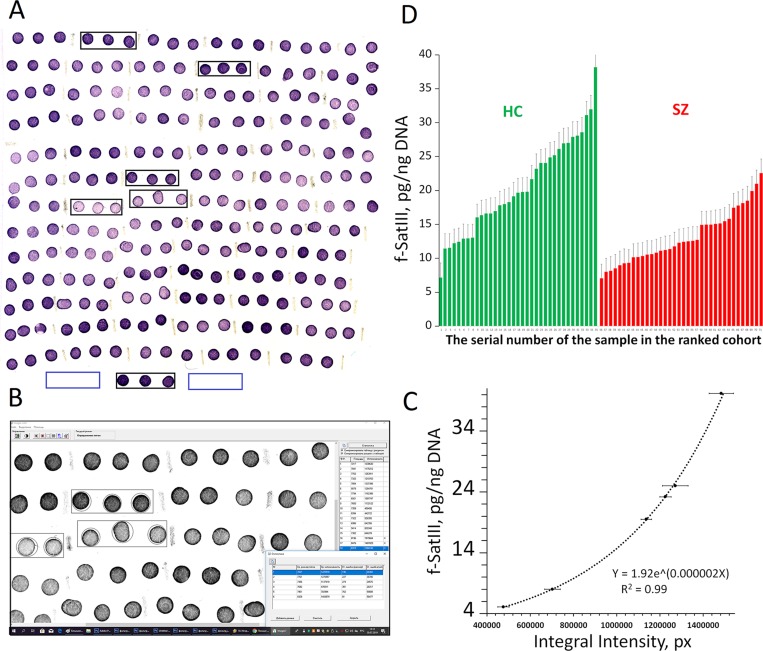
Quantitative f-SatIII determination in the human DNA by NQH. **(A)** Photo of the membrane fragments with visualized f-SatIII. 50 ng of the denatured DNA samples (three spots for each DNA sample) from the HC-group (N = 31) and SZ-group (N = 30) were applied on the filter. 50 ng of six calibration DNA samples with a known amount of f-SatIII (shown in black rectangles) were also applied to the filter. For the background signal analysis 50 ng of a non-homologous to the DNA probe DNA sample was applied (in blue rectangles). **(B)** The filter was analyzed with the “Imager 6” soft. The average integral spot intensity and the standard error were determined. **(C)** The calibration dependence of the average signal intensity on the f-SatIII content in the calibration DNA samples. The equation of the curve that was used to calculate the f-SatIII content in the analyzed DNA samples is given. **(D)** The result of the quantitative f-SatIII determination in the DNA samples from HC and the SZ-group. For clarity, the data for each group are ranked according to the f-SatIII content.

The method of NQH for f-SatIII and rDNA repeats was specified in details previously [Ershova et al., 2019b (Supplement); Ershova et al., 2019a (Supplement)]. We used this method without modifications. The experiment is shown in **Figure 1**. For the calibration, we used six standard human DNA samples with a known content of f-SatIII. This amount was previously determined by direct comparison of the content of 1.77-kb fragment (f-SatIII) in a sample of genomic DNA and in a model sample that contains a known number of molecules of the analyzed fragment. Relative standard error for NQH only was 5 ± 2%. The main contribution to the overall error of the experiment is made by the step of isolating DNA from the leukocytes. The total standard error was 11 ± 7%.

***The DNA probes**.* f-SatIII probe was a 1.77-kb cloned EcoRI fragment of human satellite DNA (Cooke and Hindley, 1979) labeled with biotin-11-2’-Deoxyuridine, 5’-Triphosphate by nick translation. Dr. H. Cook (MRC, Edinburgh, UK) kindly supplied the human chromosome lql2-specific repetitive satellite DNA probe pUC1.77.

For the detection of human rDNA pBR322-rDNA probe contains rDNA sequences (5836 bp) cloned into EcoRI site of pBR322 vector was used (Ershova et al., 2019a). The rDNA fragment cloned covers the positions from -515 to 5,321 of human rDNA (GenBank accession no. U13369).

For the detection of human TR, the probe was used: biotin-(TTAGGG)_7_. Syntol (Russia) performed the synthesis and biotin labeling of the oligo-probe.

### Fluorescent *In Situ* Hybridization

Non-stimulated human G0-lymphocytes were isolated by centrifugation in the ficoll-urography system (Paneco, Russia) from heparinized peripheral blood of men. The lymphocytes were subjected to hypotonicity (0.075 M potassium chloride solution) and were then ﬁxed [methanol/glacial acetic acid (3:1)] on glass slides. Slides with nuclei were treated with RNase A.

For the hybridization, the protocol and solutions of the company Abbott Laboratories (Abbott Laboratories, Abbott Park, IL, USA) were used with some modifications. Briefly: hybridization with bio-pUC1.77 was carried out in the thermostat ThermoBrite (StatSpin, USA). The fragment size of the probe bio-pUC1.77 was in 300–3,000 bp range as determined by electrophoresis in 1% agarose. After hybridization, biotin was detected using streptavidin conjugate with fluorescein isothiocyanate. Lymphocyte nuclei were stained with propidium iodide. The cell images were scanned using the Axioplan microscope (Opton, Germany) and REGITA 2000R digital camera (IMAGING, Canada).

### BLAST Analysis of F-SatIII Homology With the Human Genome

A 1.77-kb (f-SatIII) fragment homology analysis with the human genome was performed using the BLAST (BLASTN) programs, and the database are presented on the websites: http://www.ncbi.nlm.nih.gov and http://www.ensembl.org.

**Figure 2:**
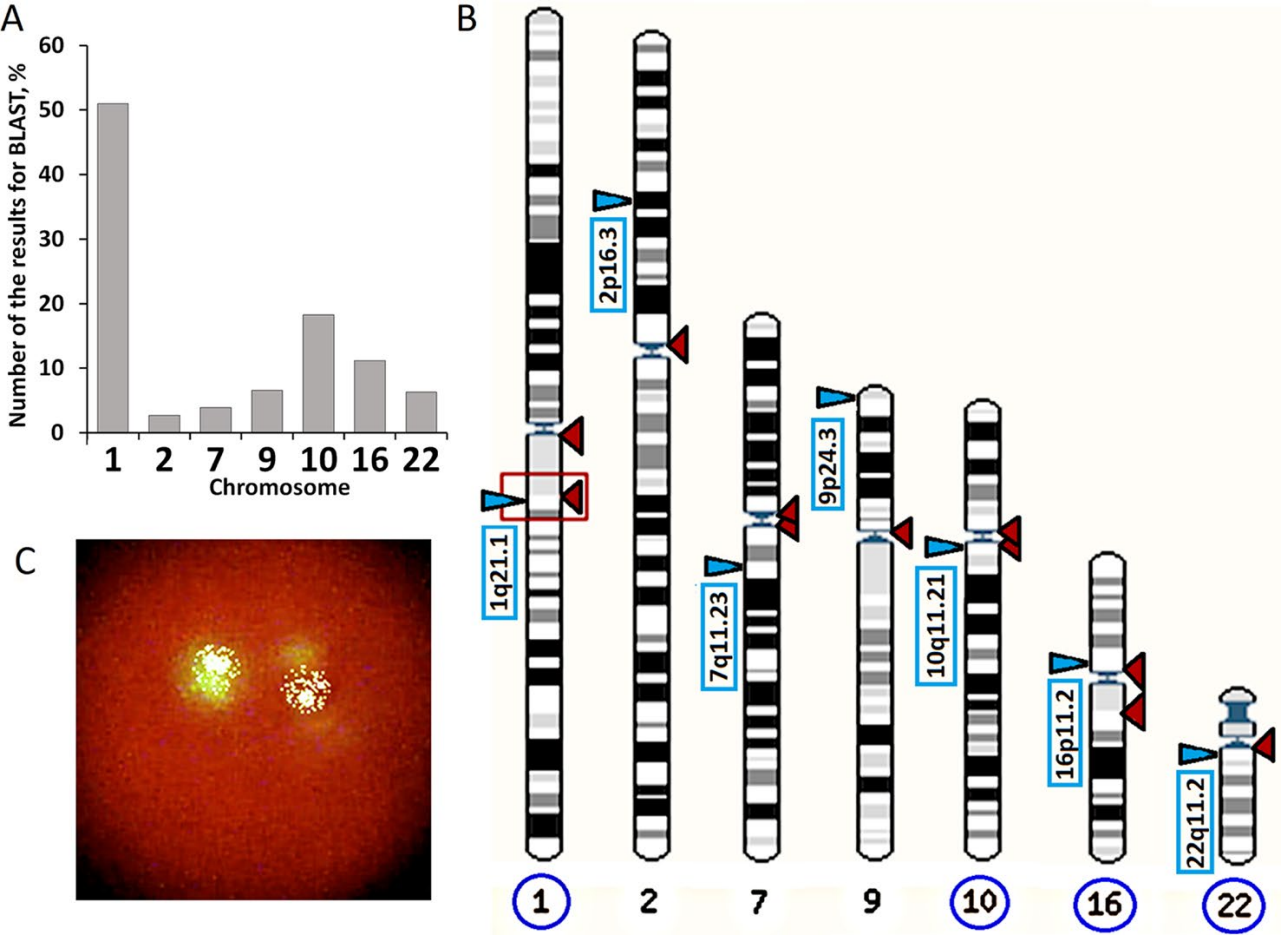
Analysis of f-SatIII in human genome with the BLAST. **(A)** BLAST analysis data. The program found 1,000 fragments from the database with more than a few copies of the f-SatIII repeat. Only fragments reliably corresponded to the certain human chromosomes were included in analysis. In the first chromosome, more than 500 fragments with dozens of f-SatIII repeat copies were found. **(B)** Distribution of f-SatIII homologous fragments on the human chromosomes (indicated by red triangles). Blue triangles indicate areas of the same chromosomes that are associated with the known literature data of CNVs-analysis of patients with mental illness. Blue circles indicate the chromosome with the close location of f-SatIII and the variable regions of the genome associated with mental disorder. **(C)** The typical example of the f-SatIII FISH analysis in the human lymphocyte nucleus. After hybridization with bio-PUC1.77 probe, biotin was detected using streptavidin conjugate with fluorescein isothiocyanate. Lymphocyte nuclei were stained with propidium iodide.

An example of the analysis is given on the page: http://www.ensembl.org/Homo_sapiens/Tools/Blast/Results?tl=j7d7io95hc6OnJQp-5514732. From 5,000 fragments, we selected 1,000 fragments that could potentially hybridize with the f-SatIII probe under severe conditions. Only those fragments that were reliably localized on the chromosomes were analyzed. The chromosome pattern in **Figure 2B** with the localization points of f-SatIII is the picture that the blast analysis gives. Only seven chromosomes contain sequences that could potentially hybridize with f-SatIII. Fragments of the Y chromosome with low homology are rare. The remaining chromosomes do not contain fragments homologous to the f-SatIII probe.

### Statistical Analysis

All the findings reported here were reproduced at least two times as independent biological replicates. The significance of the observed differences was analyzed using the non-parametric Mann–Whitney U-test (p) or the Kolmogorov–Smirnov statistics (D and α). Data were analyzed with StatPlus2007 professional software (http://www.analystsoft.com/). All p-values were two-sided and considered statistically significant at p ≤ 0.01.

## Results

### Groups of Individuals Included in the Study

We studied the content of f-SatIII in 1,241 human leukocytes DNA samples. **Table 1** shows some characteristics of the healthy volunteers group (HC, N = 401) and the group of SZ patients (SZ, N = 840).

The SZ patients were divided into four groups (**Table 1**).

1. **SZ (M-)—**first-episode drug-naïve patients.
2. **SZ (M+)—**SZ patients with a long time antipsychotics therapy.
3. **H-SZ (M+)—**SZ patients with a long time antipsychotics therapy. Patients have had documented OCs and fetal hypoxia (hypoxia group).
4. **NH-SZ (M+)—**SZ patients with a long time antipsychotics therapy. Patients have had a documented normal childbirth without any OCs and fetal hypoxia (non-hypoxia group).

The following considerations were the basis for dividing the SZ patient sample into four groups. It is known that fetal hypoxia and severe OCs that can lead to brain damage are associated with the development of mental illness (Boog, 2004; Mittal et al., 2008; Forsyth et al., 2013; Buoli et al., 2016; Giannopoulou et al., 2018). They are referred to as non-genetic factors of SZ. If pregnancy and childbirth proceeded completely without pathology, but a person fell ill with SZ, then there is a possibility that there are genetic reasons. The H-SZ (M+) and NH-SZ (M+) groups were specially formed by the employees of the MHRC to analyze the genetic and non-genetic factors that influence the onset of SZ. These groups did not differ in therapy, the presence of traumatic factors in life, and in other indicators (smoking, alcohol, obesity, social status, education).

To study the possible effect of therapy on the f-SatIII content in leukocytes, we compared two other groups: SZ (M-) and SZ (M+) from CPH1. In addition, in 93 patients of the SZ (M-) group, blood sampling was performed twice—before the treatment and a month later after the standard course of antipsychotic therapy started. Although some patients from SZ (M-) and SZ (M+) groups also reported possible OCs and fetal hypoxia, this fact was not documented and therefore was neglected in the analysis.

### Quantitative Analysis of F-SatIII Content in Human DNA

The f-SatIII content in the human DNA was analyzed by quantitative non-radioactive hybridization (NQH). The 1.77-kb cloned fragment of human satellite DNA (Cooke and Hindley, 1979) labeled with biotin (bio-pUC1.77) was used as a DNA sample.

**Figure 1** illustrating NQH analysis of 31 DNA samples of HC-group and 30 SZ (M-) samples. Six DNA calibration samples with known f-SatIII content were applied on the filter to obtain calibration dependence of the f-SatIII content on the integral hybridization signal intensity. All DNA samples were applied to the filter in the same amount—50 ng/spot. In case of using samples with different DNA concentrations, it is necessary to apply calibration samples in different quantities. An example of such an analysis is described in our previous paper (Ershova et al., 2019b).

After hybridization biotin was detected using streptavidin conjugate with alkaline phosphatase. A pair of substrates for alkaline phosphatase 5-bromo-4-chloro-3’-indolyphosphate/nitro-blue tetrazolium allows obtaining practically insoluble violet precipitate at the DNA probe hybridization sites on the filter surface. The integral intensity of the spot and the amount of hybridized DNA target are related by logarithmic dependence. The greater the amount of precipitate in the spot (the higher f-SatIII content in DNA or higher DNA target concentrations), the less pronounced the signal dependence on the amount of f -SatIII. The signal intensity may be adjusted by reducing the amount of DNA target and/or by reducing the enzymatic reaction time.

To understand which genome regions contribute to the overall signal reflecting the f-SatIII amount in the DNA, we analyzed f-SatIII homology with human genome database using BLAST programs. Analyzed repeat is localized on eight human chromosomes (**Figures 2A, B**): 1, 2, 7, 9, 10, 16, 22, and Y. Fragments of the Y chromosome with low homology are rare. The remaining chromosomes do not contain fragments homologous to the f-SatIII probe. More than half of the 1,000 homologous human f-SatIII sequences found in database are localized in the first human chromosome 1q12 region. Found in the database first chromosome fragments contain dozens of tandem repeats homologous to the f-SatIII. Fragments of the remaining chromosomes according to the database contain fewer number of the f-SatIII repeat copies.

FISH analysis data show that the main target for bio-pUC1.77 hybridization in the nucleus is 1q12 region of the first human chromosome (**Figure 2C**). The core and minor signals may be distinguished. The total intensity of the minor signals is lower than the intensity of the main signals. A comparison of BLAST analysis with FISH data allows to conclude that the DNA probe bio-pUC1.77 hybridizes mainly with the 1q12 region of the first chromosome in the total pool of DNA in the spot. However, other chromosomes that contain local areas homologous to bio-pUC1.77 also contribute to the total hybridization signal.

### Comparative Analysis of F-SatIII Content in Leukocyte DNA of Individuals of Different Schizophrenia Groups and Healthy Control Group

**Figure 3:**
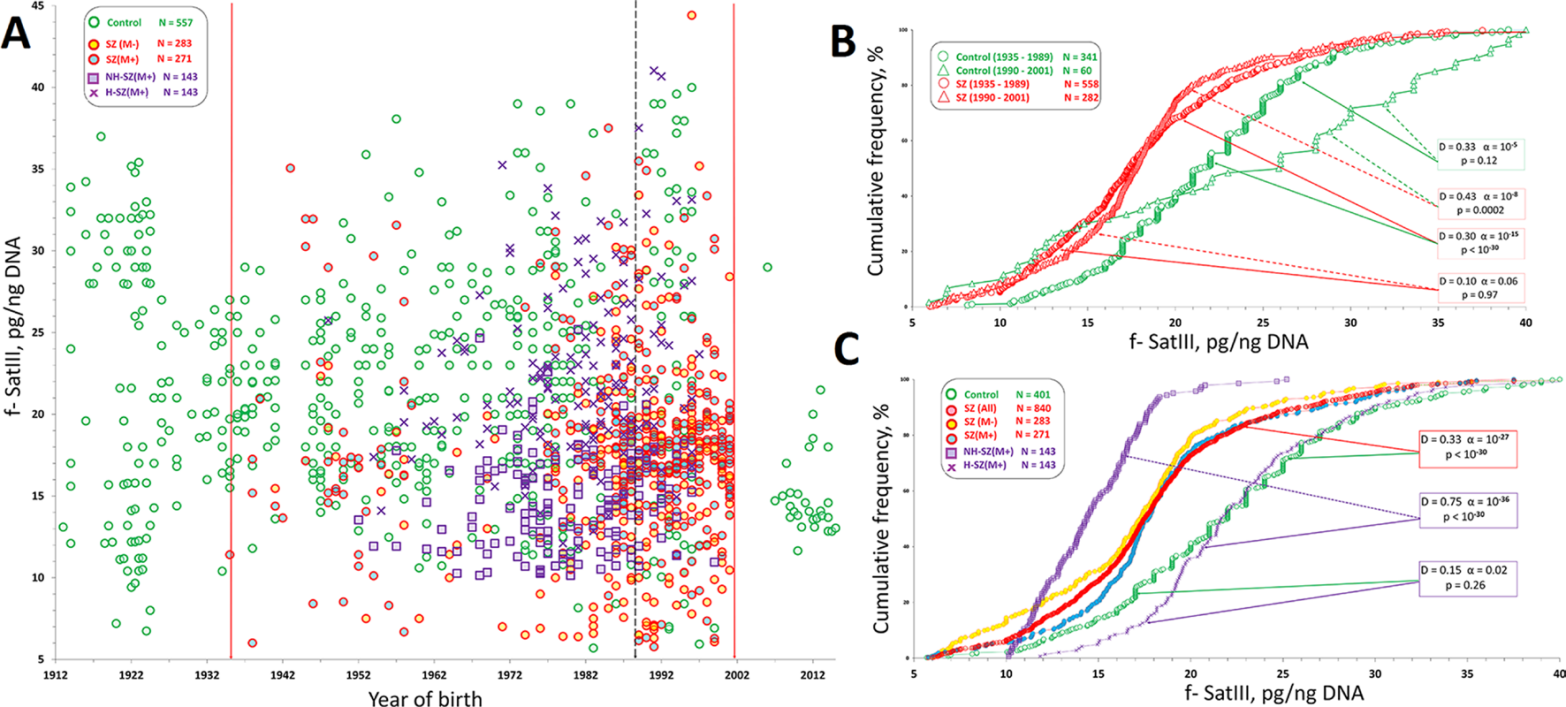
f-SatIII CNVs in HC- and SZ groups. **(A)** Dependence of the f-SatIII content in the leukocytes DNA from the year of individual’s birth. The data for 557 mentally healthy people, obtained in the previous study, are given as a control (Ershova et al., 2019b). Vertical lines limit the variation range of SZ patients’ data included in this study (1935–2001). That interval includes data for 401 individuals of the control group. Legends for four SZ patients groups are shown in the left part of the figure. **(B)** Cumulative distribution of the f-SatIII content in the leukocytes DNA of the SZ individuals and HC born in 1935–1989 and 1990–2001. The signiﬁcance of the observed differences in the f-SatIII content in the DNA samples of the groups was analyzed using non-parametric Mann–Whitney (p) and Kolmogorov–Smirnov (D and α) statistics. The arrows indicate the compared samples. **(C)** Cumulative distribution of the f-SatIII content in the leukocytes DNA of the SZ individuals and HC born between 1935 and 2001. Descriptive statistics for the f-SatIII content in the DNA samples of the groups is shown in **Table 2**.

Previously we have shown that the distribution of f-SatIII content in HC-group depends more on the individual’s year of birth than on their age (Ershova et al., 2019b). Based on these data, we presented data for SZ and HC groups on a graph that shows f-SatIII content dependence from the individual date of birth (**Figure 3A**). Date of birth in the SZ—groups varied from 1935 to 2001. This interval also includes 401 people from the control group.

**Figure 3B** shows cumulative distributions of f-SatIII content in the two cohorts of SZ patients (year of birth 1935–1989 and 1990–2001) and two cohorts of HC (year of birth 1935–1989 and 1990–2001). The distribution of f-SatIII content in the HC cohorts differ, as was shown earlier (Ershova et al., 2019b). The cohort HC (1990–2001) contains more individuals with a low repeat content and more individuals with a high repeat content (disproportionation of DNA samples by f-SatIII content). Patient cohorts did not differ in the content of f-SatIII in leukocytes. Both SZ patient cohorts differ from the corresponding HC cohorts in the low f-SatIII content. Since we did not find an association of the repeat content in SZ DNA and the year of birth, further analysis was performed for the entire SZ cohort (year of birth 1935–2001), **Figure 3C**. **Table 2** presents the descriptive statistics data.

**Table 2:** Descriptive statistics for the f-SatIII content in the DNA samples of the SZ- and HC groups.

Group	N	Mean pg/ng DNA	SD pg/ng DNA	Range pg/ng DNA	Median pg/ng DNA	Coefficient of variation
Control	401	22.0	6.7	6–40	22	0.30
SZ (All)	840	18.0	5.9	6–44	18	0.33
SZ (M-)	283	17.0	5.9	6–44	17	0.35
SZ (M+)	271	18.4	5.8	6–38	18	0.31
H-SZ (M+)	143	22.7	5.4	12–41	22	0.24
NH-SZ (M+)	143	14.7	3.0	10–25	14	0.21

The analysis of these data (**Figure 3C**) allows coming to the following conclusions.

(1) SZ patients of three groups [SZ (M-), SZ (M+), and NH-SZ (M+)] contain fewer copies of f-SatIII in the blood leukocyte genome than mentally healthy individuals (p << 10^-17^). The exception was found for patients of H-SZ (M+) group for whom the fetal H/OCs was reliably established. The content of f-SatIII in leukocytes of this group did not differ from the control group (p > 0.2).

(2) The smallest number of f-SatIII copies was observed in DNA samples of NH-SZ (M+) individuals with the documented absence of fetal H/OCs.

(3) SZ (M+) and SZ (M-) groups comparison showed that the therapy has a weak effect on the f-SatIII content in the patients DNA (p > 0.02).

### Therapy Effect on the F-SatIII Content in the Leukocytes DNA

**Figure 4:**
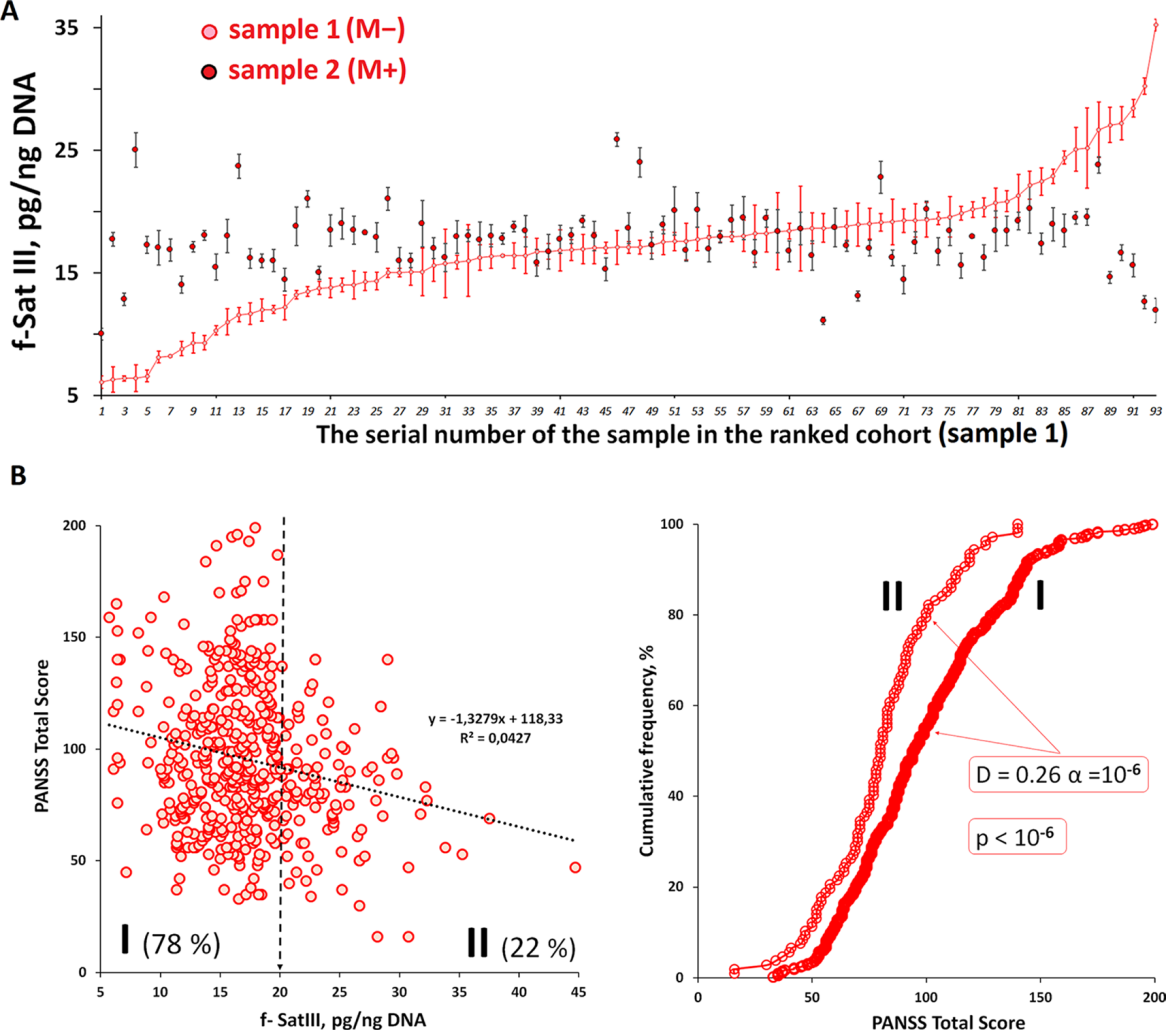
Dependence of f-SatIII content in the DNA of patients on antipsychotic therapy and the integrative PANSS score. **(A)** Change in f-SatIII content in the SZ(M-) patients DNA after a month of antipsychotics therapy. For 93 patients not taking antipsychotics at the admission to the hospital DNA was isolated from leukocytes twice—before the treatment and upon discharge from the hospital. The first test data (sample 1) are ranked for clarity. A significant increase in the f-SatIII DNA content after the treatment was found for samples with initially low f-SatIII content. F-SatIII content reduction was observed for samples with initially high repeat content. **(B)** The dependence of the integrative PANSS score on the f-SatIII content in the SZ cohort. Linear regression showed a weak negative correlation between f-SatIII and PANSS. In the subgroups of patients with low f-SatIII content (I-subgroup with f-SatIII less than 20 pg/ng DNA) the PANSS results was significantly higher than in the patients with high f-SatIII content (II-subgroup, more than 20 pg/ng). The figure on the right shows the corresponding distributions and statistics.

We investigated how the f-SatIII content in the DNA samples of 93 untreated SZ (M-) individuals changes one month after the antipsychotic therapy start (**Figure 4A**). Blood from these patients was taken twice: at admission to the hospital (sample 1) and a month after treatment started (sample 2). f-SatIII content in sample 1 given in **Figure 4A** is ranked for clarity. In the patients with initially very low f-SatIII (6–12 pg/ng DNA) we found an increase in this repeat content in the course of antipsychotic therapy. Conversely, in the DNA of patients with a relatively high initial f-SatIII content (25–35 pg/ng DNA) the repeat content later decreases. Comparison of these two samples showed that the treatment in general does not lead to the change in the DNA f-SatIII content (D = -0.17, α = 0.11; p > 0.2). The variation coefficient of this value in samples 2 is reduced twice compared to samples 1 (from 0.31 to 0.15). The f-SatIII content also didn’t depend on the type of used neuroleptics (p > 0.1).

### F-SatIII Content Dependence on the Mental Disorder Severity

The PANSS scale was used in both institutions for the patients’ mental state assessment. It was performed at the discharge from the hospital. For the SZ patients we found a negative association between f-SatIII content and PANSS results (**Figure 4B**). In the subgroups of patients with low f-SatIII content (I-subgroup with f-SatIII less than 20 pg/ng DNA) the PANSS results was significantly higher than in the patients with high f-SatIII content (II-subgroup, more than 20 pg/ng).

### F-SatIII Content Variability in the Different Human Brain Structures

**Figure 5:**
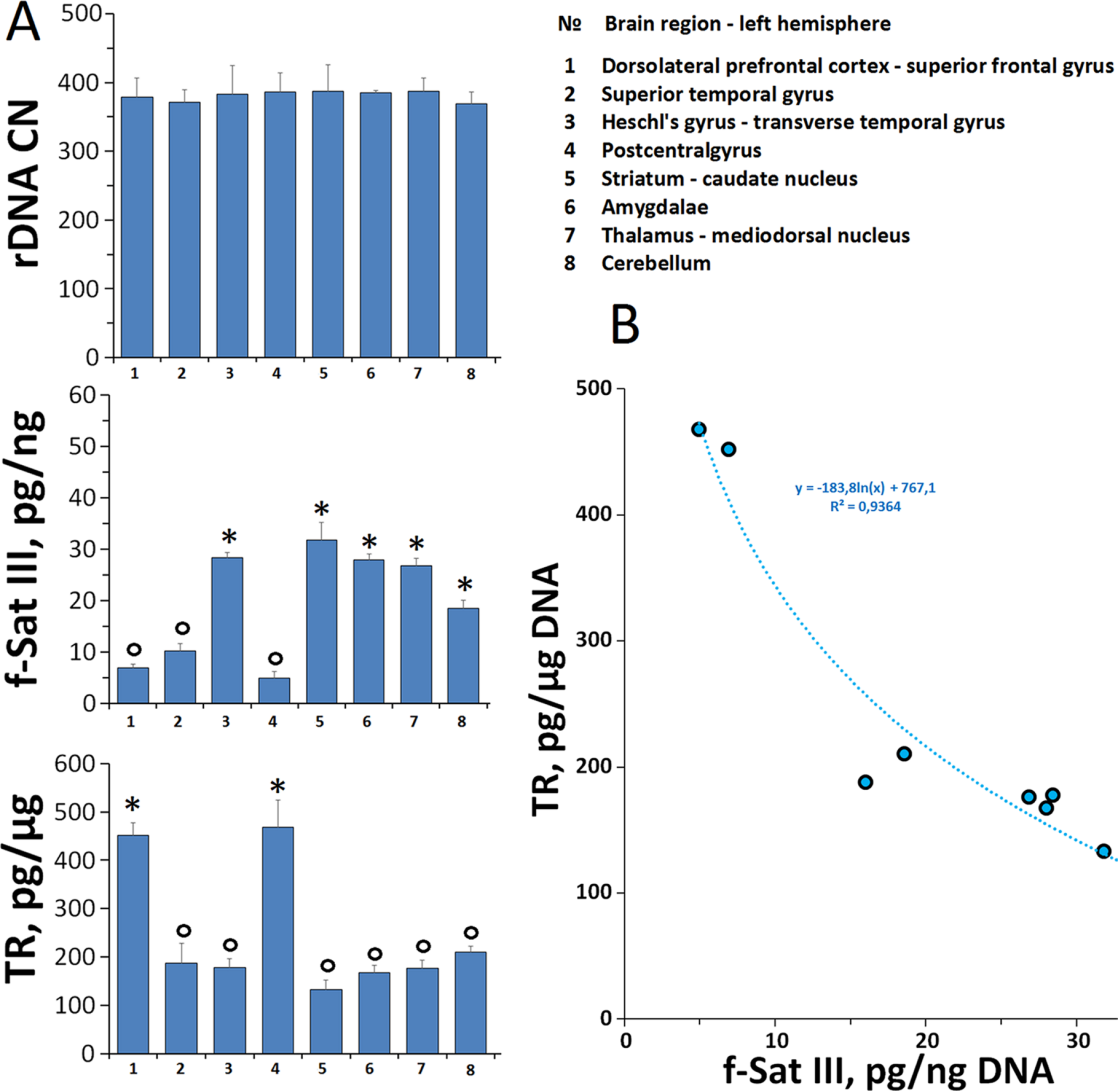
f-SatIII CNVs in the DNA samples isolated from the different brain regions. **(A)** DNA was isolated from eight brain regions (1–8, the designation on the right table) of the patient with a continuous paranoid schizophrenia. For the same DNA samples the contents of three tandem repeats (rDNA, f-SatIII and telomere repeat) were determined using NQH method. (o) —samples with the low repeat content, (*)—the samples with a high repeat content. **(B)** The dependence of the telomere repeat contents on the f-SatIII content.

We have previously found that the population of cultured human skin fibroblasts is heterogeneous by the f-SatIII content (Ershova et al., 2019b). To test whether quantitative f-SatIII polymorphism is possible in cells of one human brain tissue, we studied the f-SatIII content in DNA isolated from paranoid SZ patient different eight brain regions (**Figure 5**).

For comparison, we also analyzed two other tandem repeats—ribosomal repeat (rDNA) and telomere repeat (TR) using the NQH method.

**rDNA.** The rDNA content in all eight DNA samples was similar (p < 0.05). The SZ patient genome contains 381 ± 7 rDNA copies.

**f-SatIII.** DNA samples were divided into two subgroups, which differ significantly by the f-SatIII content (p < 0.05). Subgroup SZ(o) contains 7.3 ± 2.5 pg f-SatIII/ng DNA (samples 1, 2, 4). Subgroup SZ(*) contains 3.7 times more f-SatIII repeat (26.8 ± 4.8 pg/ng DNA), samples 3, 5–8. Thus, in the various regions of the SZ patient brain we found polymorphism in the f-SatIII content. The maximum content of f-SatIII (32 pg/ng DNA) was observed in striatum caudate nucleus (sample 5), the minimum (5 pg/ng DNA)—in postcentral gyrus (sample 4).

**TR.** The TR content of the DNA isolated from different parts of the brain also varies significantly. We compared the f-SatIII and TR content in the same DNA samples (**Figure 5**). The content of these DNA repeats negatively correlated with each other: the more f-SatIII was in the sample, the lower was the TR content. Earlier in the analysis of the replication aging of cultured skin fibroblasts we found the same relation—an increase in the f-SatIII content with a decrease in TR during the cultured cells aging (Ershova et al., 2019b).

## Discussion

### Reduced F-SatIII Content in Schizophrenia Patients’ Blood Leukocytes

We have previously showed that oxidative stress can induce an increase in the number of the f-SatIII copies or a loss of a part of the f-SatIII copies in the cultured human cells. The effect depends on the intensity of stress and the individual characteristics of the cultured cells. These two responses to stress were also observed in the leukocytes of irradiated people, the elderly population and those who were born during a period of social upheaval (Ershova et al., 2019b).

It is known that SZ patients experience oxidative stress (Emiliani et al., 2014; Sertan Copoglu et al., 2015; Smaga et al., 2015; Hardingham and Do, 2016; Koga et al., 2016; Barron et al., 2017; Maas et al., 2017; Patel et al., 2017) and stress associated with the hospitalizations and impaired social adaptation (Howes and Murray, 2014). Planning this study, we assumed that there would be a disproportion by the f-SatIII content between SZ patients compared with the HC group of the same year of birth. Especially high variability we expected to see in the group of SZ patients who experienced fetal hypoxia with serious OCs.

However, we found another effect. The content of f-SatIII in human leukocytes decreases in a following manner:

HC≈H−SZ(M+)>SZ(M+)≈SZ(M−)>NH−SZ(M+)

The lowest f-SatIII content was observed in leukocytes of NH-SZ (M+) patients with documented normal delivery without intrauterine H/OCs. The highest f-SatIII content was found in the patients with documented H/OCs. SZ (M+) and SZ (M-) groups also included patients who experienced and did not experience H/OCs. Accordingly, the average repeat content was higher than in the NH-SZ (M+) group and lower than in the H-SZ (M+) group or in the control.

Fetal H/OCs are attributed to non-genetic risk factors for SZ (Boog, 2004; Mittal et al., 2008; Forsyth et al., 2013; Buoli et al., 2016; Giannopoulou et al., 2018). Although some authors suggest that such stressors provoke SZ only in the case of the genetic predispositions. Significant differences in the leukocytes DNA f-SatIII content of patient belonging to the groups H-SZ (M+) and NH-SZ (M+) may possibly be explained by various contributions of genetic and non-genetic pathogenic factors. Apparently, the NH-SZ (M+) group includes the largest number of patients with the genetic SZ predisposition.

**Figure 6:**
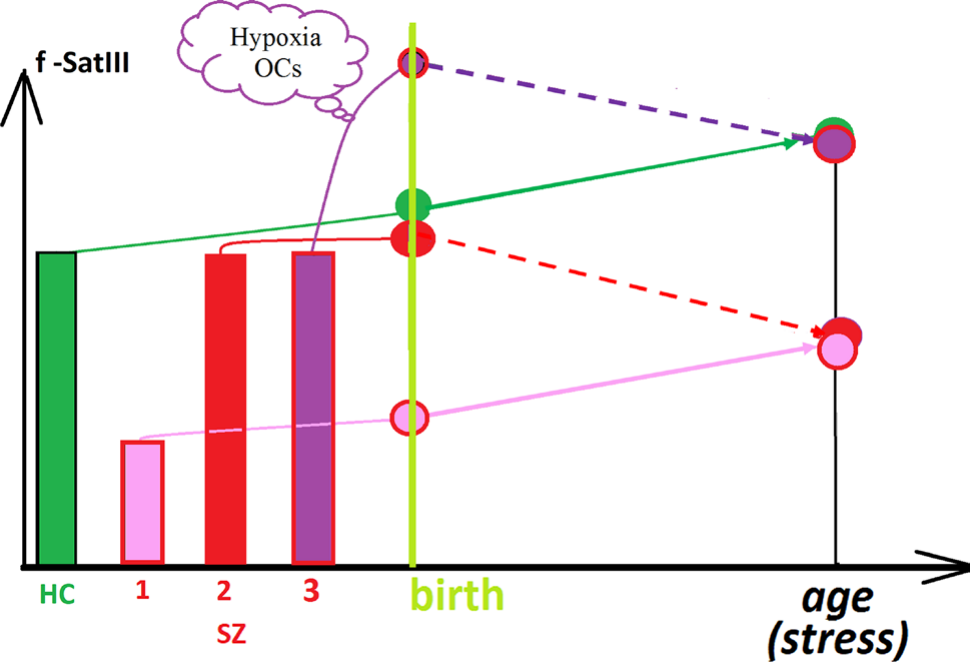
A hypothetical scheme illustrating the changes in the f-SatIII content during human life. HC: healthy individual. During the aging the content of f-SatIII usually increases. Patient SZ-1: deletion of the genome regions containing f-SatIII (low repeat content). During the aging the content of f-SatIII increases. Patient SZ-2: normal f-SatIII content in the genome; during the aging the content of the repeat decreases. Patient SZ-3 [H-SZ (M+)]: normal f-SatIII content in the genome at the beginning of embryogenesis. Hypoxia and OCs stimulate an increase in repeat content. During the aging the content of the repeat decreases.

Further studies are needed to determine the real causes and consequences of the low f-SatIII content in the leukocytes DNA typical for the most patients with SZ. Only some speculative considerations may be possible now. Two most likely hypotheses can be put forward (**Figure 6**).

1. Congenital low f-SatIII content in the genomes of SZ patients may be a consequence of deletions associated with the development of pathology [example SZ-1 (pink color), **Figure 6**]. The 1q12 site which contains a lot of f-SatIII repeats is one of the most unstable regions of human genome with often rearrangements, for example, in cancer. Perhaps other f-SatIII-containing regions are also characterized by reduced stability. It is interesting to note that in several CNVs associated with mental disorder (Bassett et al., 2010; Vassos et al., 2010; Kirov et al., 2014; Marshall et al., 2017; Zhuo et al., 2017; Kushima et al., 2018; Kendall et al., 2019; Warland et al., 2019), four CNVs (**Figure 2B**) were localized in the same chromosome regions as f-SatIII. Perhaps, f-SatIII is a marker indicating the deletion of the significant for the normal brain functioning regions of the genome. The f-SatIII location in the genome may indicate other, yet unknown chromosome regions associated with mental disorder.
2. The low f-SatIII content in the SZ patients may be a consequence of chronic oxidative stress associated with this disease. The genomes of SZ patients may initially contain the normal amounts of f-SatIII and respond to the stress reducing the repeats copies number (example SZ-2, **Figure 6**). H/OCs can increase f-SatIII content in the SZ genome, but the development of the disease further induces a decrease in this repeat. Perhaps that is why the genomes of the H-SZ (M+) group contain amounts of DNA comparable to control (example SZ-3, **Figure 6**), while most of the genomes of healthy people respond to chronic oxidative stress (IR) by increasing the f-SatIII content (Ershova et al., 2019b).

The low number of f-SatIII copies in human cells may be linked with the mental disorder. We found a negative correlation between f-SatIII content in the DNA and PANSS index (**Figure 4B**). Low f-SatIII content in DNA of patients was associated with more pronounced mental disorder. Changes in the highly repetitive sequence content may cause gene expression disorders in the nuclei of the brain cells. This effect was found for another tandem repeat—ribosomal repeat (rDNA). It was shown that a change in the rDNA content in the nucleus leads to the changes in the large number of genes expression level (Paredes and Maggert, 2009; Paredes et al., 2011). It is interesting to note that in SZ we found an increase in the rDNA copies number in the patients’ genomes (Chestkov et al., 2018). It is also known that during human cells stress response and in G1 and S cell cycle phases 1q12 regions containing f-SatIII repeat are moving from the surface of the nucleus inside and are localized close to the nucleolus, where ribosomal repeats are located (Léger et al., 1994; Ermakov et al., 2009). It is possible that a decrease in the number of f-SatIII copies is an adaptive genome response to an increased number of ribosomal repeats.

Earlier we found that the genomes of children aged 3–10 years (born between 2005–2015) contain low amounts of f-SatIII compared to the genomes of young people (over 17 years). The content of f-SatIII in the group of children is comparable with the repeat content in the NH-SZ (M+) group of adult patients. We propose that in the puberty period of healthy people there is an increase in the white blood cells f-SatIII content. The molecular mechanism of that effect may be impaired in SZ patients’ blood leukocytes. In the future, a comparative analysis of the f-SatIII content in the embryonic tissues, leukocytes of newborns, of children in the first years of life and adolescents is of a great interest. Such analysis will allow to make a more unambiguous conclusions about the reasons for the low f-SatIII content in the SZ patients DNA.

### Antipsychotic Therapy Modulates the F-SatIII Content in SZ Patients’ Blood Leukocytes

First-episode drug-naïve patients of the SZ (M-) group differ a little by their leukocytes f-SatIII content from patients of the SZ (M+) group who took antipsychotics for a long time (**Figure 3C**). A month therapy course increased the f–SatIII content in leukocytes of patients from the SZ (M-) group with low repeat content and a decreased in those with a high repeat content. Further research is needed to understand these changes reasons. Obviously, the leukocytes of human blood are heterogeneous by f-SatIII content. Previously, we have shown the cells f-SatIII heterogeneity for the cultured human skin fibroblasts. Perhaps the therapy changes the proportions of high and low DNA f-SatIII containing blood cells.

### Areas of the Schizophrenic Patient Brain Are Heterogeneous by F-SatIII Content

The variability of human cells f-SatIII repeat content was confirmed on the cells isolated from different regions of a paranoid SZ patient postmortem brain. For greater reliability, we determined the content of three tandem repeats—ribosomal, f-SatIII and TRs in the DNA isolated from eight brain regions. Analysis of TR and f-SatIII in brain structures in a patient with SZ shows a high degree of variability in the content of these genome regions in the cortical and subcortical structures of the brain. At the same time, as we have already shown for fibroblasts (Ershova et al., 2019b), the content of these two repeats in the DNA of brain cells is negatively correlated with each other: the more f-SatIII in the sample, the lower the content of TR. The founded phenomena requires further study and comparison of the data with the results of healthy individuals analysis, clarification of the possible DNA variability source (neurons, glia) in order to determine the possible role of TR and f-SatIII polymorphism in the different brain structures in the SZ.

Recently, several authors have shown that in response to stresses, pericentromeric heterochromatic domains of the human genome change their epigenetic status and acquire euchromatic features (Suzuki et al., 2002; Jolly et al., 2004; Rizzi et al., 2004; Valgardsdottir et al., 2005; Jolly and Lakhotia, 2006; Sengupta et al., 2009). This is accompanied by the transcriptional activation of these regions with the production of SatIII RNAs. Transcription of Satellite III non-coding RNAs is a general stress response in human cells (Valgardsdottir et al., 2008). SatIII RNAs seem to be stable components of nuclear stress body, suggesting that they may have an important role in the recruitment of a number of RNA binding proteins to the bodies. It was also shown that human chromosome 1 (1q12) satellite III DNA is decondensed, demethylated and transcribed in senescent cells and in carcinoma cells (Enukashvily et al., 2007). A decrease in SatIII RNAs due to a decrease in the total number of SatIII DNAs can potentially affect the ability of body cells to respond to stress. Possibly, a low amount of SatIII DNAs in the genomes is one of the possible causes of oxidative stress in the body of patients with SZ.

## Conclusion

The content of the f-SatIII repeat in leukocytes of the most patients with SZ is significantly reduced compared to the healthy control. Two hypotheses were put forward: (1) the low content of the repeat is a genetic feature of SZ; and/or (2) the genomes of the SZ patients respond to chronic oxidative stress reducing the repeats copies number.

## Data Availability Statement

All datasets generated for this study are included in the article/supplementary material.

## Ethics Statement

The investigation was carried out in accordance with the latest version of the Helsinki Declaration and approved by the Regional Ethics Committee of Research Centre for Medical Genetics. All participants signed an informed written consent to participate after the procedures had been completely explained.

## Author Contributions

SVK, VG, SIK, and NV designed the study. NZ, LB, EJ, TL, and GK examined and selected patients for the study, performed analysis using a scale PANSS and provided the human blood samples. EE, OA, AYM, and AVM performed the experiments. RV performed the statistical analysis, created a computer database for SZ- and HC-groups and program “Imager 6.0.” SVK, PU, and NV wrote the initial draft and translated the manuscript to English. All the authors participated in critical revision and approved the manuscript before submission.

## Funding

The Russian Science Foundation (grant no. 18-15-00437) supported this research.

## Conflict of Interest

The authors declare that the research was conducted in the absence of any commercial or financial relationships that could be construed as a potential conflict of interest.
